# Living cover crops are associated with higher earthworm abundance and biomass in a subtropical citrus orchard

**DOI:** 10.3389/fpls.2026.1841226

**Published:** 2026-09-08

**Authors:** Chengcheng Zeng, Zhongyi Li, Hongqin Tang, Lirong Su, Tianming Su, Yuefeng Yu, Hai Liang, Wenbin Dong, Caihui Wei, Tieguang He

**Affiliations:** 1 Agricultural Resources and Environmental Research Institute, Guangxi Academy of Agricultural Sciences, Nanning, China; 2 Guangxi Key Laboratory of Arable Land Conservation, Nanning, China

**Keywords:** *Arachis pintoi*, citrus orchard, cover crop management, earthworm, *Indigofera hendecaphylla*, soil nutrients

## Abstract

Living cover crops are increasingly used in orchards, but evidence linking cover-crop identity to earthworm indicators under subtropical conditions remains limited. This cross-sectional field comparison was based on one sampling event in November 2025. We compared perennial peanut (*Arachis pintoi*) and *Indigofera hendecaphylla* Jacq. ex Poir. with a weedy control and measured community-level earthworm abundance and fresh biomass, plant-derived inputs, and selected soil properties at 0–10 and 10–20 cm. Mixed-effects models accounting for the randomized block design detected treatment- and depth-associated differences in earthworm abundance and biomass. At 0–10 cm, *I. hendecaphylla* had the highest earthworm abundance (156.00 ± 40.92 ind. m⁻²) and fresh biomass (2.408 ± 0.365 g m⁻²). Across 0–20 cm, total abundance and fresh biomass were 78.1% and 93.3% higher, respectively, under *A. pintoi* than under the weedy control, and 208.2% and 218.3% higher under *I. hendecaphylla*. *I. hendecaphylla* also had the highest litter standing crop (625.89 ± 32.45 g fresh mass m⁻²) and the highest topsoil organic matter, total nitrogen, and alkali-hydrolyzable nitrogen. Correlation and ordination analyses summarized associations with surface litter and nutrient-related variables but did not establish independent drivers or causality. At this single November sampling event, both living covers were associated with higher earthworm abundance and fresh biomass, with the strongest association under *I. hendecaphylla*. Temporal persistence requires multi-season sampling, dry-mass measurements, and species or ecological-group identification.

## 1 Introduction

Soil biodiversity is a fundamental component of agroecosystem functioning, and belowground organisms play central roles in organic matter turnover, nutrient cycling, soil aggregation, and water regulation (Bardgett and van der Putten, 2014). Earthworms are widely recognized as key ecosystem engineers because their feeding, burrowing, and casting activities strongly influence soil structure, microbial processes, and the delivery of ecosystem services (Blouin et al., 2013). Consequently, earthworm abundance and biomass are frequently used as practical indicators of soil biological health and sustainable soil use (Schon et al., 2023).

In perennial orchard systems, soil biological communities are highly sensitive to orchard-floor management between tree rows. Repeated disturbance, bare-soil maintenance, herbicide use, and intensive chemical inputs may reduce soil habitat quality for soil macrofauna, whereas inter-row vegetation cover or residue retention can improve soil moisture, moderate temperature fluctuations, increase organic inputs, and provide more favorable microhabitats for soil organisms (Przybyłko et al., 2022). Recent syntheses indicate that alternative agricultural practices generally benefit soil biodiversity, and among the major practices evaluated, cover crops are one of the few approaches that are consistently neutral to positive across multiple soil taxonomic and functional groups (Cozim-Melges et al., 2025). In orchards specifically, mulching or cover crop-derived residues have been shown to increase soil moisture and to promote earthworm abundance and mass (Webber et al., 2022). Cover crops are also increasingly recognized as important drivers of soil ecosystem services, including soil biodiversity maintenance, nutrient cycling, and improved soil physical conditions (Blanco-Canqui et al., 2015; Blanco-Canqui, 2022; Yousefi et al., 2024). Citrus-orchard studies likewise show that inter-row cover crops can modify soil nutrient cycling and microbial communities, with several responses concentrated in the 0–10 cm soil layer (Castellano-Hinojosa et al., 2023a, 2023b). In subtropical citrus orchards, ground-cover management has also been linked to changes in arbuscular mycorrhizal fungal communities and ecosystem multifunctionality, highlighting the ecological importance of ground-cover composition and functional diversity (Feng et al., 2024; Zou et al., 2026). A citrus-focused review further identifies cover-crop selection, soil microbial responses, weed regulation, and nutrient management as central considerations in orchard cover-crop systems (Silwana et al., 2023).

Earthworm responses to land use and vegetation management are, however, strongly context dependent. In orchard and other perennial systems, vegetation cover management can be expected to shape earthworm communities through its effects on food resources, microclimate, and soil physical conditions (Blanco-Canqui, 2022; Webber et al., 2022). At the same time, broader soil biodiversity assessments continue to emphasize that locally grounded field data remain insufficient in many regions and production systems, particularly for linking management practices to biologically meaningful indicators (Guerra et al., 2024).

Species identity is especially relevant because living covers differ in growth form, rooting distribution, residue production, and nitrogen acquisition, which can alter the quantity and spatial distribution of resources available to soil biota. We selected *A. pintoi* and *I. hendecaphylla* because both are perennial warm-climate legumes suitable for persistent orchard-floor cover, but they can provide different patterns of plant-derived input. *A. pintoi* is a low-growing, prostrate legume used as a living mulch; previous studies show that it can contribute biologically fixed nitrogen and increase microbially available carbon, providing a basis for expecting associations through continuous ground protection, rhizosphere activity, and root-derived inputs (Duda et al., 2003; Rose et al., 2019). *I. hendecaphylla* is a creeping, densely rooted legume historically used as a green-manure and cover crop in humid perennial plantations; it forms a persistent surface cover, suppresses weeds, and can produce nitrogen-rich green biomass (Sunarno, 1997). The two species were evaluated here as alternative single-species inter-row covers; they were not co-planted and were not selected because they co-occur naturally at the study site. Comparing them therefore addressed whether two contrasting management options were associated with different plant inputs, soil conditions, and community-level earthworm responses.

Living cover crops have been increasingly adopted between orchard rows as a strategy to improve soil quality and ecological sustainability. However, their associations with earthworm abundance and biomass remain insufficiently documented under many orchard conditions, particularly in subtropical citrus orchards. We therefore compared two leguminous inter-row covers, *A. pintoi* and *I. hendecaphylla*, with a weedy control and separated earthworm responses between the 0–10 and 10–20 cm layers. We tested whether treatment identity was associated with community-level earthworm abundance and fresh biomass at one November sampling event and whether any association was stronger in surface soil. The study was designed as a field-based, cross-sectional comparison and not as evidence of temporal persistence or causality.

## 2 Materials and methods

### 2.1 Study site and experimental design

The study was conducted in a *Citrus reticulata* cv. ‘Wogan’ orchard at the experimental base of the Guangxi Academy of Agricultural Sciences, Nanning, Guangxi, China (22.834°N, 108.282°E; 79 m above sea level). The study area has a subtropical monsoon climate, with a mean annual temperature of 21.6 °C and mean annual precipitation ranging from 1304.0 to 1453.4 mm. The orchard was approximately 5 years old at the time of sampling, with a planting spacing of 4 m × 2 m. The soil type was classified as calcareous soil. The 0–20 cm soil had a sandy loam texture, with 10.8% clay, 31.9% silt, and 57.3% sand, as determined by particle-size analysis.

Before establishment of the cover-crop treatments in March 2024, weeds were removed manually, the inter-row soil was subjected to shallow tillage, and the removed plant material was taken out of the orchard. These practices were applied uniformly before randomization. From treatment establishment to sampling in November 2025, no herbicide or soil-applied insecticide application was recorded within the experimental inter-rows. Routine agronomic inputs applied to the citrus-tree rows, when used, were uniform across all plots and were not treatment-specific.

Three inter-row treatments were established in March 2024: weedy control, *Arachis pintoi* Krapov. & W.C. Greg., and *Indigofera hendecaphylla* Jacq. ex Poir. The weedy control was dominated by naturally occurring weeds. The experiment was arranged in a randomized complete block design with four blocks. Within each block, the three treatments were randomly assigned to one plot each. Each plot measured approximately 4 m × 3 m (approximately 12 m²), and adjacent plots within a block were separated by approximately 0.5 m bare-soil buffer strips. Sampling quadrats were placed at least 0.5 m from plot borders. Soil, plant, and earthworm samples were collected once in November 2025 and separated into 0–10 and 10–20 cm layers. Accordingly, the experiment supports treatment comparisons for that sampling event but not inference about seasonal or year-round treatment stability.

### 2.2 Earthworm sampling and biomass determination

Earthworms were sampled during the morning in November 2025, on the same date as soil and plant sampling. November was selected as a late active-season sampling window in subtropical southern China. At a subtropical plantation in nearby Heshan, monthly surface-cast production exceeded 80 g m⁻² from April through November but was below 30 g m⁻² from December through March, indicating substantially lower winter activity (Wang et al., 2021). This regional activity pattern contextualizes the choice of November but does not make one date representative of an annual mean or seasonal maximum. Sampling used a 0.25 m² quadrat in each replicate plot and followed the hand-sorting principle in ISO 23611-1:2018, with minor modifications (International Organization for Standardization, 2018). Soil was excavated and sorted separately at 0–10 and 10–20 cm, and abundance and biomass were converted to a 1 m² basis.

For each soil layer, all recovered earthworms, including juveniles and adults, were pooled for abundance and fresh-biomass measurements. Adhering soil was removed with clean water, individuals were blotted with absorbent paper, and total fresh mass was recorded immediately. Mean individual mass was total biomass divided by abundance. Total 0–20 cm abundance and biomass were calculated by summing the paired layer values within each replicate. Earthworms recovered from 10–20 cm were retained as a separate depth record. However, no preserved voucher material was available for retrospective species identification or assignment to epigeic, endogeic, or anecic categories. Results are therefore restricted to community-level abundance, fresh biomass, and mean individual mass.

### 2.3 Plant residue and biomass measurements

Litter standing crop, fresh aboveground biomass, and fresh root biomass were measured at the same November 2025 sampling event. All plots followed the same routine mowing regime, and sampling occurred immediately before the next scheduled mowing. Exact dates and frequency of earlier mowing were not retained in the available field record; this constraint is stated because mowing history can affect litter standing crop. Litter and living aboveground plant material were collected separately from a 0.25 m² quadrat, weighed immediately, and expressed as g fresh mass m⁻².

Fresh root biomass was measured separately at 0–10 and 10–20 cm from the same sampling units. Roots were washed over a fine sieve, cleared of visible mineral particles and non-root debris, blotted, weighed immediately, and expressed as g fresh mass m⁻². No biomass subsamples were oven-dried, so dry-matter conversion or treatment-specific moisture-correction factors were not available. The plant variables therefore represent fresh-mass pools at the sampling event rather than dry-matter production or carbon input.

### 2.4 Soil physicochemical analysis

Soil samples were collected from the same plots and depths at the same November 2025 sampling event. Gravimetric soil moisture was measured on fresh subsamples by oven drying and is reported as a contemporaneous indicator of field moisture. Plot-level rainfall during the preceding 7–14 days was not recorded, so a reliable antecedent-rainfall total cannot be reported. Except for fresh subsamples used for moisture and microbial biomass measurements, soils were air-dried, gently ground, and passed through a 2-mm sieve. Soil pH was measured potentiometrically using a portable pH meter (PHB-4, INESA, Shanghai, China). Soil organic matter (OM) was determined by potassium dichromate oxidation titration, and soil organic carbon (OC) was estimated from OM using the conventional factor of 1.724 (Nelson and Sommers, 1982; Bao, 2000). Total nitrogen (TN) was determined using an elemental analyzer (Elementar Vario EL III, Elementar Analysensysteme GmbH, Langenselbold, Germany). Total phosphorus (TP), total potassium (TK), and available potassium (AK) were determined by ICP-OES (Agilent 5800, Agilent Technologies, Santa Clara, CA, USA). Alkali-hydrolyzable nitrogen (AN) was measured by the alkali diffusion method, and available phosphorus (AP) by the molybdenum–antimony colorimetric method using a Multiskan GO 1510 microplate reader (Thermo Fisher Scientific, Waltham, MA, USA) (Bao, 2000). Microbial biomass carbon (MBC) and nitrogen (MBN) were determined by chloroform fumigation–extraction, with extractable C and N quantified using a multi N/C 3300 analyzer (Analytik Jena, Jena, Germany) (Brookes et al., 1985; Vance et al., 1987).

### 2.5 Statistical analysis

All analyses were performed in R 4.3.0 (R Core Team, 2023). Linear mixed-effects models accounted for the randomized complete block design and repeated depth measurements. For depth-resolved earthworm variables, treatment, depth, and their interaction were fixed effects, and block and plot nested within block were random intercepts. For total 0–20 cm abundance and biomass, treatment was fixed and block was random. Pairwise comparisons used estimated marginal means with Tukey adjustment at P < 0.05. Residual-versus-fitted and Q–Q plots, Shapiro–Wilk tests, and Levene’s tests were used to assess assumptions. Earthworm abundance was log10(x + 1)-transformed and fresh root biomass was log-transformed when this improved residual diagnostics. Means ± SD are presented on the original scale. Pearson correlations were used after inspection of bivariate scatterplots. Variables were centered and scaled before PCA and RDA. PCA summarized multivariate separation in the 0–10 cm layer. RDA used earthworm abundance, biomass, and mean individual mass as responses and litter standing crop, root biomass, soil moisture, pH, and AN as constraints. Overall RDA significance was evaluated with 999 permutations. The surface-layer analysis contained 12 observations and five intercorrelated constraints; consequently, inference was restricted to the overall permutation test, and biplot arrows were treated as descriptive orientations rather than evidence that any variable made a significant independent contribution. Percentage change was ((treatment mean − control mean)/control mean) × 100. Hedges’ g was calculated as J × ((Mtreatment − Mcontrol)/Spooled), with J = 1 − 3/(4df − 1) (Hedges and Olkin, 1985). A Poisson generalized linear model including treatment, depth, their interaction, and block was used as a sensitivity analysis for abundance. A negative binomial model did not improve fit because the estimated dispersion parameter approached zero.

## 3 Results

### 3.1 Treatment and soil depth were associated with differences in earthworm abundance and biomass

**Figure 1:**
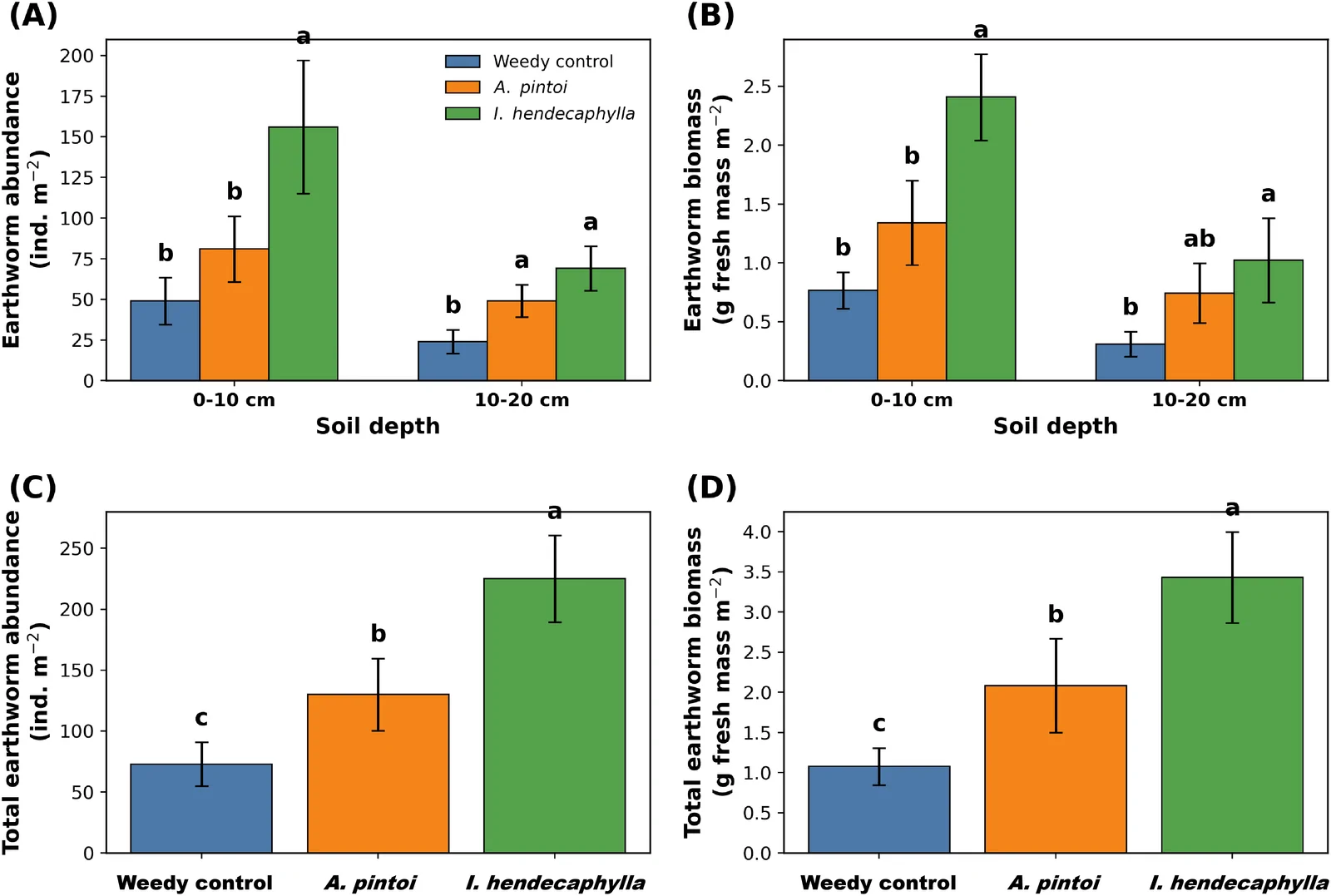
Treatment-associated differences in earthworm abundance and fresh biomass across soil depths. **(A)** Earthworm abundance in the 0–10 and 10–20 cm soil layers under the weedy control, *Arachis pintoi*, and *Indigofera hendecaphylla* treatments; **(B)** earthworm biomass in the 0–10 and 10–20 cm soil layers under the same treatments; **(C)** total earthworm abundance in the 0–20 cm soil profile; **(D)** total earthworm biomass in the 0–20 cm soil profile. Abundance and biomass are expressed on a 1 m² basis; biomass is reported as fresh mass. Values are means ± SD (n = 4). Different lowercase letters indicate significant differences among treatments within the same soil layer or soil profile according to Tukey-adjusted pairwise comparisons at P < 0.05.

Mixed-effects models showed that log-transformed earthworm abundance differed by treatment (χ² = 49.47, df = 2, P < 0.001) and depth (χ² = 22.48, df = 1, P < 0.001), whereas the treatment × depth interaction was not significant (χ² = 2.34, df = 2, P = 0.310). Earthworm fresh biomass differed by treatment (χ² = 136.22, df = 2, P < 0.001), depth (χ² = 10.24, df = 1, P = 0.001), and their interaction (χ² = 24.67, df = 2, P < 0.001). Mean individual mass differed by depth (χ² = 5.56, df = 1, P = 0.018) but not by treatment (χ² = 0.20, df = 2, P = 0.904) or the interaction (χ² = 1.00, df = 2, P = 0.607) (**Figure 1**; **Supplementary Tables S1**–**S3**). At 0–10 cm, abundance was 156.00 ± 40.92, 81.00 ± 20.23, and 49.00 ± 14.38 ind. m⁻² under *I. hendecaphylla*, *A. pintoi*, and the weedy control, respectively; fresh biomass was 2.408 ± 0.365, 1.340 ± 0.359, and 0.767 ± 0.154 g m⁻². At 10–20 cm, the corresponding abundances were 69.00 ± 13.61, 49.00 ± 10.00, and 24.00 ± 7.30 ind. m⁻², and fresh biomasses were 1.022 ± 0.358, 0.743 ± 0.254, and 0.310 ± 0.106 g m⁻². Thus, individuals were recovered from both layers, although the larger treatment-associated differences occurred in surface soil at this sampling event. The Poisson sensitivity analysis yielded the same inferential pattern: treatment (χ² = 83.01, df = 2, P < 0.001) and depth (χ² = 49.41, df = 1, P < 0.001) were significant, whereas their interaction was not (χ² = 1.82, df = 2, P = 0.402).

Across 0–20 cm, total abundance averaged 73.00 ± 18.00, 130.00 ± 29.67, and 225.00 ± 35.53 ind. m⁻² in the weedy control, *A. pintoi*, and *I. hendecaphylla* treatments, respectively. Total fresh biomass followed the same ranking at 1.078 ± 0.231, 2.083 ± 0.584, and 3.430 ± 0.567 g m⁻² (**Figure 1**; **Supplementary Table S2**). Block-adjusted models detected treatment differences in total abundance (χ² = 69.69, df = 2, P < 0.001) and total biomass (χ² = 156.41, df = 2, P < 0.001).

### 3.2 Magnitude of observed treatment differences

**Figure 2:**
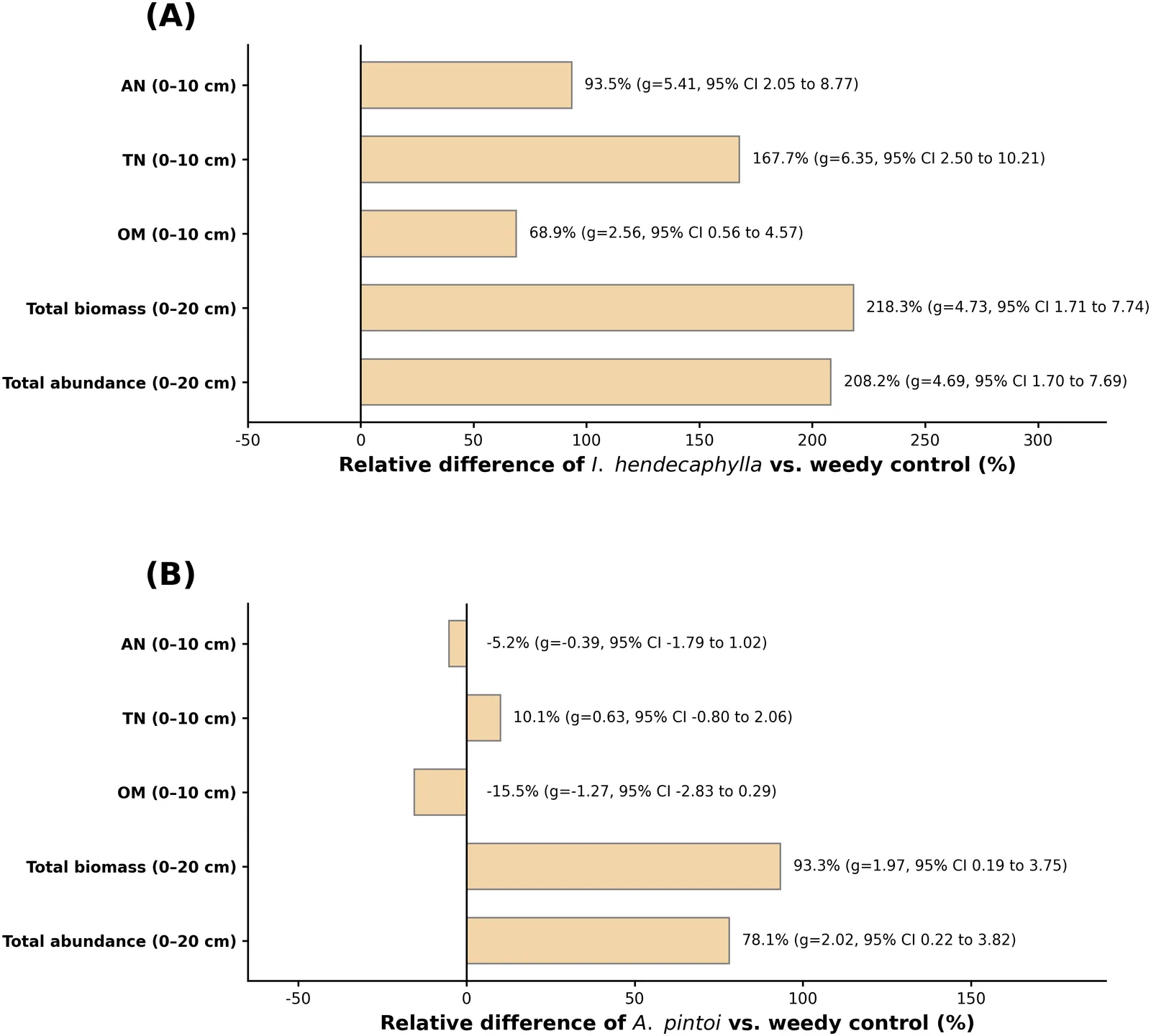
Relative differences and standardized effect sizes for living cover crops compared with the weedy control. **(A)** Percentage change and Hedges’ g values with approximate 95% confidence intervals for *Indigofera hendecaphylla* relative to the weedy control; **(B)** percentage change and Hedges’ g values with approximate 95% confidence intervals for *Arachis pintoi* relative to the weedy control. Effect sizes were calculated for total earthworm abundance and total fresh biomass in the 0–20 cm soil profile, and for soil organic matter (OM), total nitrogen (TN), and alkali-hydrolyzable nitrogen (AN) in the 0–10 cm layer. Positive values indicate higher values relative to the weedy control, whereas negative values indicate lower values.

Standardized effect sizes summarized the magnitude of treatment contrasts relative to the weedy control (**Figure 2**; **Supplementary Table S4**). At the November sampling event, total 0–20 cm abundance and fresh biomass were 78.1% and 93.3% higher under *A. pintoi*, with Hedges’ g values of 2.02 (95% CI, 0.22 to 3.82) and 1.97 (95% CI, 0.19 to 3.75), respectively. Under *I. hendecaphylla*, total abundance and fresh biomass were 208.2% and 218.3% higher, with g values of 4.69 (95% CI, 1.70 to 7.69) and 4.73 (95% CI, 1.71 to 7.74), respectively.

Topsoil nutrient contrasts differed between the two living covers (**Figure 2**). Relative to the weedy control, *A. pintoi* had 10.1% higher TN (g = 0.63), 15.5% lower OM (g = −1.27), and 5.2% lower AN (g = −0.39). In contrast, *I. hendecaphylla* had 68.9%, 167.7%, and 93.5% higher OM, TN, and AN, respectively, with g ranging from 2.56 to 6.35. This contrast, together with plant-input data, motivated the species-specific interpretation developed below.

### 3.3 Plant-derived inputs and soil properties differed among treatments

**Figure 3:**
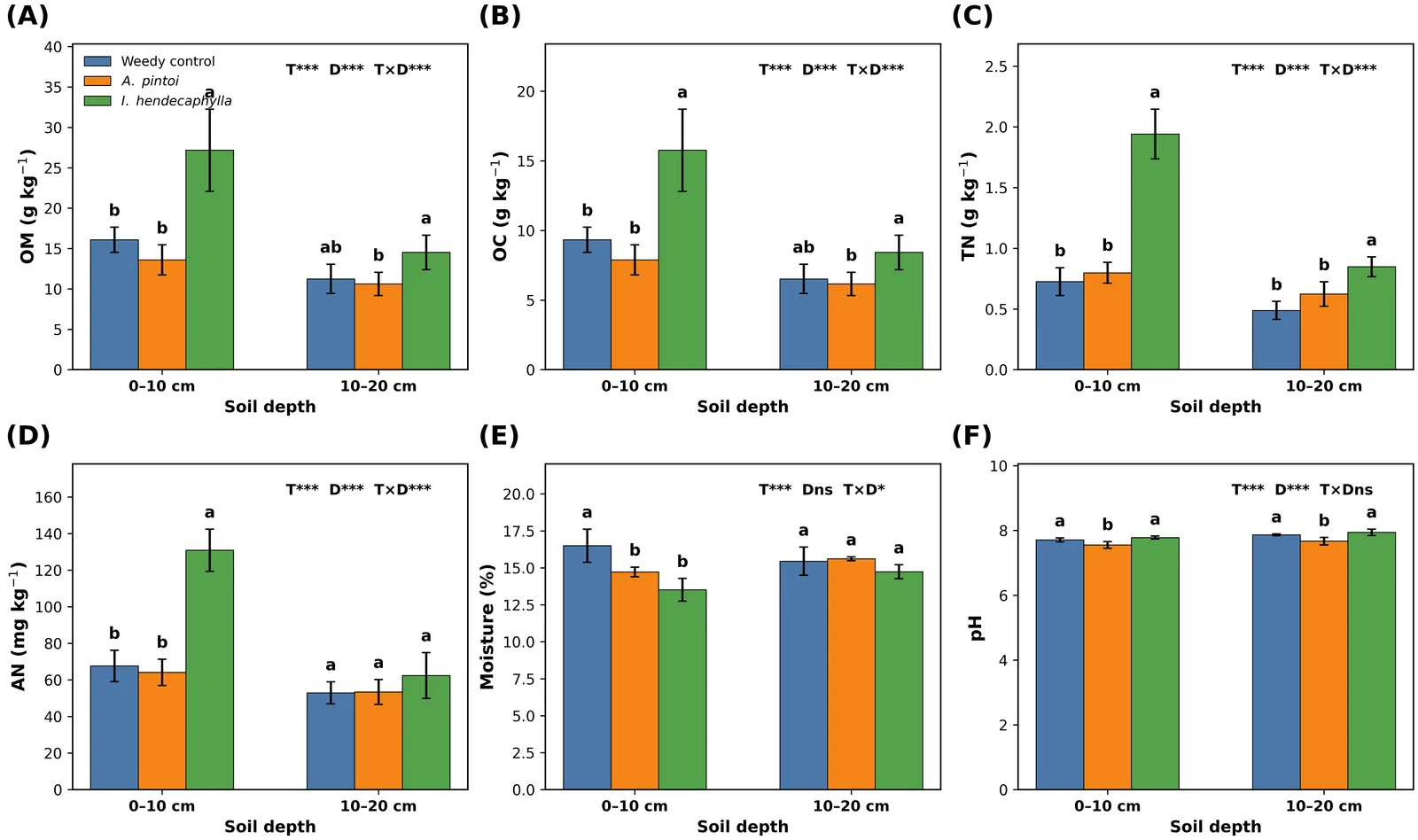
Treatment-associated differences in selected soil physicochemical properties across soil depths. **(A)** Soil organic matter (OM); **(B)** soil organic carbon (OC); **(C)** total nitrogen (TN); **(D)** alkali-hydrolyzable nitrogen (AN); **(E)** soil moisture; **(F)** pH. Values are means ± SD (n = 4). Different lowercase letters indicate significant differences among treatments within the same soil layer according to Tukey-adjusted pairwise comparisons at P < 0.05. Significance results for cover crop treatment (T), soil depth (D), and treatment × depth interaction (T×D) are shown in each panel.

Plant-derived inputs differed markedly among treatments (**Figure 3**; **Supplementary Tables S5**–**S7**). At 0–10 cm, litter standing crop was highest under *I. hendecaphylla* (625.89 ± 32.45 g fresh mass m⁻²), followed by *A. pintoi* (458.83 ± 54.18) and the weedy control (291.34 ± 81.69). Conversely, *A. pintoi* had the greatest fresh aboveground biomass (2750.87 ± 104.38 g m⁻²) and root biomass at both 0–10 cm (3417.25 ± 247.87 g m⁻²) and 10–20 cm (678.17 ± 59.00 g m⁻²). Thus, the treatment with the greatest living biomass was not the treatment with the greatest litter standing crop, topsoil nutrient concentrations, or earthworm response.

For the variables shown in **Figure 3**, block-adjusted analyses detected treatment differences in OM, OC, TN, AN, soil moisture, and pH. Depth differences occurred for OM, OC, TN, AN, and pH but not soil moisture. Treatment × depth interactions were detected for OM, OC, TN, AN, and soil moisture (**Supplementary Tables S6**, **S7**), indicating that the treatment-associated contrasts were concentrated mainly in the surface layer.

At 0–10 cm, *I. hendecaphylla* had the highest OM (27.17 ± 5.09 g kg⁻¹) and AN (130.86 ± 11.54 mg kg⁻¹), compared with 16.09 ± 1.56 g kg⁻¹ and 67.63 ± 8.58 mg kg⁻¹ in the weedy control and 13.59 ± 1.85 g kg⁻¹ and 64.11 ± 7.18 mg kg⁻¹ under *A. pintoi* (**Figure 3**; **Supplementary Table S6**). Concurrent topsoil moisture was highest in the weedy control (16.50 ± 1.12%), intermediate under *A. pintoi* (14.72 ± 0.33%), and lowest under *I. hendecaphylla* (13.62 ± 0.77%). Consequently, *A. pintoi* combined the greatest living aboveground and root fresh biomass with comparatively low litter accumulation and no corresponding topsoil OM or AN enrichment, whereas *I. hendecaphylla* combined the greatest litter standing crop with the most nutrient-enriched topsoil.

### 3.4 Associations among earthworm, plant-input, and soil variables

**Figure 4:**
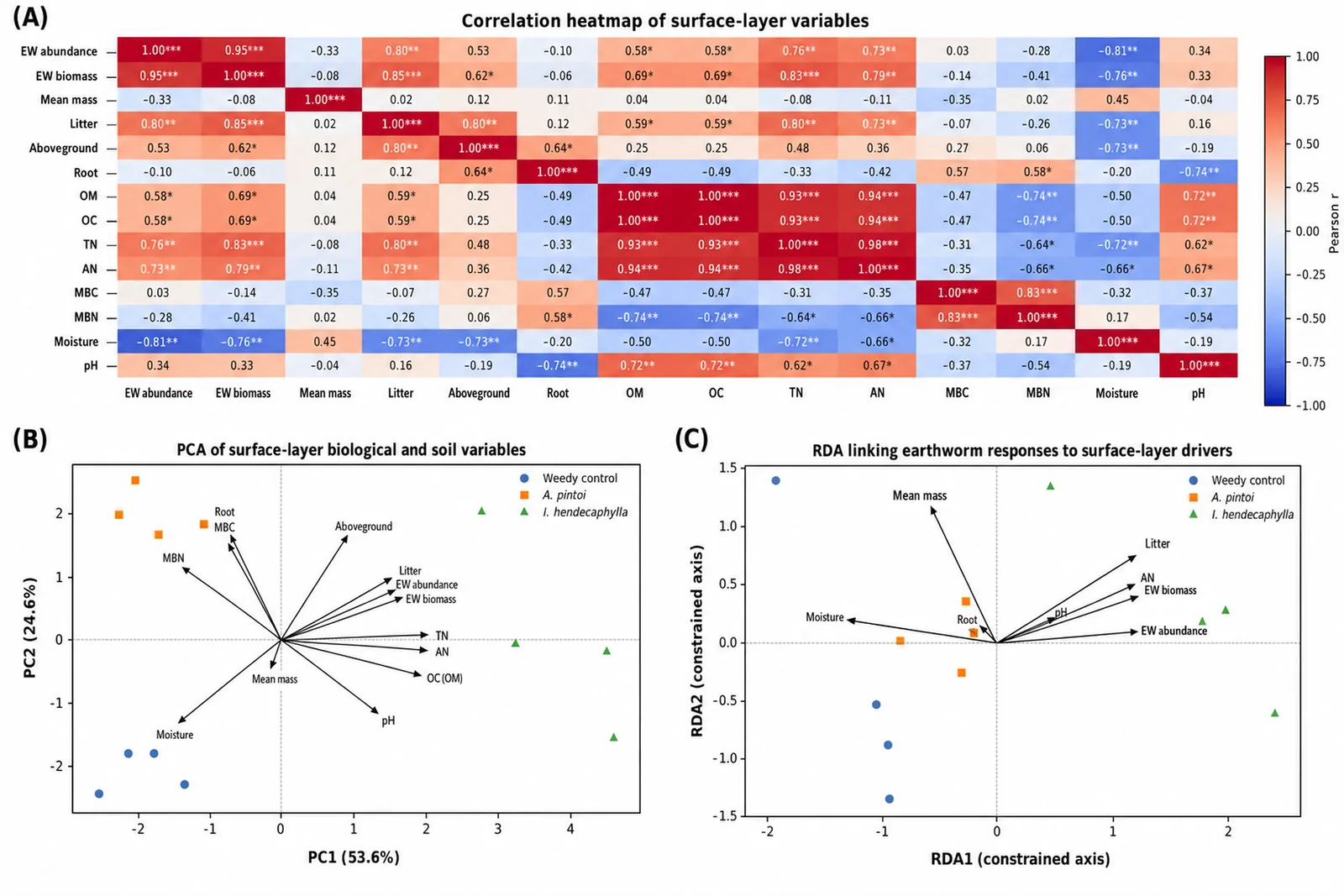
Integrated correlation and multivariate ordination analyses of earthworm, plant, and soil variables under different cover crop treatments. **(A)** Enlarged correlation heatmap of earthworm, plant-derived input, and soil variables measured in the 0–10 cm layer. Variable labels were abbreviated to improve readability: EW abundance, earthworm abundance; EW biomass, total earthworm biomass; Mean mass, mean individual earthworm body mass; Litter, litter standing crop; Aboveground, fresh aboveground biomass; Root, fresh root biomass; OM, soil organic matter; OC, soil organic carbon; TN, total nitrogen; AN, alkali-hydrolyzable nitrogen; MBC, microbial biomass carbon; MBN, microbial biomass nitrogen; Moisture, soil moisture content. Color intensity indicates Pearson correlation coefficients, and asterisks indicate significant correlations (P < 0.05, P < 0.01, P < 0.001). **(B)** Principal component analysis (PCA) of centered and scaled biological and soil variables measured in the 0–10 cm soil layer. PC1 and PC2 explained 53.6% and 24.6% of the total variance, respectively. **(C)** Redundancy analysis (RDA) linking earthworm responses to selected environmental variables in the 0–10 cm layer. RDA1 and RDA2 explained 74.0% and 25.6% of the constrained variance, respectively. The overall model was significant (adjusted R² = 0.586; permutation P = 0.0096); arrows are descriptive biplot orientations and are not evidence of significant independent contributions. In panels **(B, C)**, symbols represent individual samples from the weedy control, *A. pintoi*, and *I. hendecaphylla* treatments. Arrows indicate the direction and relative orientation of biological and soil variables in the ordination space.

In the 0–10 cm layer, earthworm abundance was positively correlated with litter standing crop (r = 0.80), TN (r = 0.76), AN (r = 0.73), and OM (r = 0.58), and negatively correlated with soil moisture (r = −0.81) (**Figure 4A**). Fresh biomass showed similar correlations with litter (r = 0.85), TN (r = 0.83), AN (r = 0.79), OM (r = 0.69), and moisture (r = −0.76). The negative moisture correlations coincided with treatment structure: the weedy control had the highest mean topsoil moisture and lowest earthworm values, whereas *I. hendecaphylla* had the lowest mean moisture and highest earthworm values. This cross-sectional pattern should therefore not be interpreted as evidence that lower moisture promotes earthworms; it may reflect treatment-associated covariance among vegetation, resources, moisture, and earthworm responses.

PCA summarized clear multivariate separation among treatments, with PC1 and PC2 explaining 53.6% and 24.6% of total surface-layer variance (**Figure 4B**). *I. hendecaphylla* samples were positioned with higher earthworm abundance, biomass, litter, OM, OC, TN, and AN; weedy-control samples were positioned with higher moisture; and *A. pintoi* samples were generally intermediate. These positions describe covariance in the observed samples rather than causal pathways.

The overall RDA model was significant (R² = 0.774; adjusted R² = 0.586; permutation P = 0.0096), and RDA1 and RDA2 accounted for 74.0% and 25.6% of constrained variance, respectively (**Figure 4C**). Earthworm abundance and biomass were oriented toward litter and AN and away from moisture in the biplot. However, the constraints were intercorrelated and the analysis included only 12 samples; arrow orientation was therefore used descriptively and was not interpreted as evidence that any one variable contributed significantly and independently. Supplementary ordination for 10–20 cm showed a different, weaker separation pattern (**Supplementary Figure S1**).

## 4 Discussion

At the single November 2025 sampling event, both living cover crops were associated with higher community-level earthworm abundance and fresh biomass than the weedy control, particularly at 0–10 cm, and the association was strongest under *I. hendecaphylla*. The same treatment also had the largest litter standing crop and the highest topsoil OM, TN, and AN. This co-occurrence is consistent with literature linking orchard-floor residues and biologically active surface soil to earthworm habitat and food resources (Blanco-Canqui, 2022; Webber et al., 2022), but the present cross-sectional correlations and ordinations cannot identify a causal pathway.

The depth pattern narrows, but does not resolve, the ecological interpretation. Epigeic earthworms live and feed mainly in surface litter, anecic earthworms occupy mineral-soil burrows while feeding substantially on surface litter, and endogeic earthworms live and feed mainly within mineral soil (Bottinelli et al., 2020; Huang et al., 2020). The alignment between surface litter and the largest 0–10 cm response is compatible with a prominent contribution from litter-responsive epigeic or anecic taxa. Nevertheless, earthworms were also recovered at 10–20 cm in every treatment (24–69 ind. m⁻²), demonstrating mineral-soil occupancy at sampling and leaving open a contribution from endogeic or anecic taxa. Depth alone is not diagnostic of ecological category, so the pooled data cannot distinguish an epigeic-dominated response from a broader community shift.

The contrast between the two legumes is the most informative species-level result. *A. pintoi* produced the greatest living aboveground and root fresh biomass but had less standing litter, lower topsoil OM and AN than the control, and an intermediate earthworm response. *I. hendecaphylla* combined the greatest litter standing crop with the highest topsoil nutrient concentrations and earthworm values. This pattern suggests that the amount retained at the soil surface, residue turnover, and substrate quality may align more closely with earthworm responses than living biomass alone. Earthworm-mediated litter mass loss has been reported to be much greater for litter with C/N <20 than for litter with C/N >20 (136.1% versus 28.2% in a global synthesis), and the response differs among ecological groups (Huang et al., 2020). Experiments also show that residue chemistry and lignin content can modify earthworm-associated decomposition and soil C responses (Jiang et al., 2018; Zheng et al., 2018). However, we did not measure litter C/N, lignin:N, cellulose, polyphenols, microbial conditioning, or decomposition rate, and no archived litter remained for retrospective analysis. These variables are therefore plausible hypotheses, not demonstrated explanations. A direct test should pair litter chemistry with litterbag mass loss, soil microclimate, and species- or ecological-group-resolved earthworm sampling across seasons.

Seasonal context further limits generalization. In a nearby subtropical plantation, monthly surface-cast production was >80 g m⁻² from April through November and <30 g m⁻² from December through March, a greater than 2.7-fold contrast in an activity proxy between the active and winter periods (Wang et al., 2021). That result supports interpreting November as the end of a relatively active window, but it does not establish that our November abundance or biomass equals an annual mean or peak. The inverse moisture correlation in our data also should not be generalized ecologically because it tracked treatment separation and was based on one date. Repeated sampling across wet, dry, warm, and cool periods is required to determine whether treatment rankings persist or change with season.

Four additional limitations are important. First, no preserved specimens were available for species or ecological-group identification, so community composition and diversity responses remain unknown. Second, earthworm, litter, aboveground, and root biomass were measured only as fresh mass. No dry-mass subsamples or moisture-correction factors were available, and antecedent 7–14-day plot-level rainfall was not recorded. Although concurrent gravimetric soil moisture was measured, treatment comparisons of plant fresh mass could still be confounded if the cover species differed in tissue water content. Third, exact earlier mowing dates and frequency could not be reconstructed, even though plots shared the same management regime and sampling preceded the next mowing. Fourth, the surface-layer RDA used five correlated constraints with only 12 observations. Its significant overall test supports multivariate association, but variable arrows and pairwise correlations do not demonstrate significant independent contributions. These limitations restrict the study to a transparent cross-sectional comparison rather than a mechanistic or temporally general treatment test.

For orchard management, *I. hendecaphylla* is the more promising of the two covers for the measured soil biological indicators at this November sampling event, because it combined the largest litter standing crop with the highest community-level earthworm abundance and fresh biomass. This result is not yet sufficient for a general recommendation. Multi-season and multi-year comparisons should also quantify dry biomass, litter quality and decomposition, earthworm composition, citrus yield and root competition, water use, mowing requirements, and other management costs or trade-offs before one species is preferred for subtropical citrus orchards.

In conclusion, living cover crops were associated with higher community-level earthworm abundance and fresh biomass than the weedy control at one November sampling event, and *I. hendecaphylla* showed the strongest association. The simultaneous litter and nutrient pattern identify a testable hypothesis for future work, not proof of a litter-mediated mechanism, long-term biodiversity improvement, or restoration.

## Data Availability

The original contributions presented in the study are included in the article/**Supplementary Material**. Further inquiries can be directed to the corresponding authors.

## Supplementary material

Supplementary Figure S1 Multivariate ordination of biological and soil variables in the 10–20 cm soil layer under different cover crop treatments. **(A)** Principal component analysis (PCA) based on earthworm, root biomass, and soil variables measured in the 10–20 cm layer. PC1 and PC2 explained 42.3% and 21.2% of total variance, respectively; **(B)** RDA linking earthworm responses to selected environmental variables in the 10–20 cm layer. RDA1 and RDA2 explained 85.9% and 13.7% of constrained variance, respectively. Symbols represent individual samples. Arrows show descriptive directions of covariance and should not be interpreted as independent effects.
